# Early Development of Fig (*Ficus carica* L.) Root and Shoot Using Different Propagation Medium and Cutting Types

**DOI:** 10.21315/tlsr2021.32.1.5

**Published:** 2021-03-31

**Authors:** Muhammad Syufihuddin Shamsuddin, Rozilawati Shahari, Che Nurul Aini Che Amri, Nur Shuhada Tajudin, Mohd. Radzali Mispan, Mohd Syahmi Salleh

**Affiliations:** 1 Department of Plant Science, Kulliyyah of Science, International Islamic University Malaysia, 25200 Kuantan, Pahang, Malaysia; 2 Research Institute and Agricultural Development Malaysia, 43400 Serdang, Selangor, Malaysia

**Keywords:** *Ficus carica* L., Propogation Medium, Cutting Types, Root, Shoot, *Ficus carica* L., Medium Penanaman, Jenis Keratan Batang, Pertumbuhan Akar dan Pucuk

## Abstract

This study aimed at determining the effects of propagation medium and cutting types on the early growth performance of fig (*Ficus carica L.*) root and shoot. The experiment was conducted at the Glasshouse and Nursery Complex (GNC), International Islamic University Malaysia (IIUM). The split-plot design was employed with the main plot (propagation medium) and sub-plot (types of cutting). The propagation medium were sand:topsoil (1:3) (M1), topsoil:peat:sawdust (1:1:1) (M2) and peat:perlite (1:1) (M3). Two types of cutting were semi-hardwood (C1) and hardwood (C2). As a result, there were a significant effect of propagation medium on measured parameters. This study revealed that the most effective propagation medium and cutting types for the propagation of fig were a combination of peat and perlite at 1:1 ratio (M3) and hardwood cutting (C2), respectively as evidenced by significantly higher root and shoot growth quality as compared to other treatments.

## Abstract (ms)

Kajian ini bertujuan untuk menentukan kesan media dan jenis keratan pada perkembangan awal akar dan pucuk pokok tin (*Ficus carica* L.). Eksperimen ini telah dijalankan di Glasshouse and Nursery Complex (GNC), Universiti Islam Antarabangsa Malaysia (UIAM). Reka bentuk “split plot” telah digunakan dengan plot utama (media) dan sub plot (keratan batang). Medium yang digunakan ialah pasir:tanah lapisan atas (1:3) (M1), tanah lapisan atas:lumut gambut:habuk papan (1:1:1) (M2) dan lumut gambut:perlit (1:1) (M3). Dua jenis keratan yang digunakan ialah keratan batang separa matang (C1) dan keratan batang matang (C2). Hasil kajian menunjukkan bahawa media memberikan kesan signifikan terhadap jisim segar keseluruhan pucuk, jisim kering akar, bilangan akar yang baru muncul, bilangan akar tertiari dan panjang akar sekunder terpanjang. Manakala, keratan batang keras menunjukkan kesan signifikan yang lebih tinggi terhadap jisim segar dan kering akar, bilangan akar tertiari, panjang akar primer dan sekunder yang terpanjang. Keputusan dari kajian ini menunjukkan bahawa medium dan jenis keratan terbaik ialah gabungan lumut gambut dan perlite pada kadar 1:1 (M3) dan keratan batang matang (C2) berdasarkan kepada pertumbuhan akar dan pucuk yang lebih tinggi secara signifikan berbanding rawatan yang lain.

**Highlights**

- The survival rate of hardwood cuttings was the highest compared to semi-hardwood cuttings.
- 75 hardwood cutting survived when grown in a medium consisting of 50% perlite and 50% sand.
- Early growth rate of *Ficus carica* increased by the usage of peat and perlite as propagation medium and hardwood cuttings as propagation materials.

## INTRODUCTION

Fig (*Ficus carica* L.) is known to have high nutritional and medicinal values, including anti-oxidant, anti-cancer and anti-bacterial properties. Fig contains approximately 1.3% to 3.6% protein, 9.5% to 52.9% total sugar, 45 kcal to 300 kcal energy, 5.2% to 28.6% glucose, and 4.1% to 22.7% fructose per 100 g fresh and dry fruits (Aksoy *et al.* 1998). According to Manago (2006), a fig is native to western Asia and has been cultivated in the Mediterranean and North Africa for thousands of years. Although this plant requires the warm and dry temperature to grow, it is adaptable to different climates in both sub-tropical and temperate regions. Recently, fig cultivation has spread to countries in Southeast Asia such as Thailand, Indonesia and Malaysia.

Currently, air layering method is adopted in fig cultivation by smallholder farmers in Malaysia, but it is not advisable. It is because the method may have a higher success rate on early fig development, but it cannot produce many seedlings due to a delicate mother plant. There is also a cultivation method by tissue culture for commercial purposes. However, this method requires a massive cost and will take a long time to produce fig seedlings. Besides, seedlings produced from tissue culture may face survival problems during the acclimatisation process.

Apart from that, the stem-cutting propagation method may also be utilised in fig cultivation. Nevertheless, this propagation method is time-consuming and produces a lower survival rate of the cuttings (approximately 40%–50%), making it not practical to adopted (Darwesh *et al.* 2014). Propagation by cutting involves the initial root formation of the stem. Root plays a vital role in plants, especially anchorages, as well as in the transport of nutrients and water throughout cutting during early growth. Proper media selection is, therefore, needed to enhance healthy root development. Therefore, this study was conducted to determine the effect of different planting medium and cutting types on an early shoot and root development of *Ficus carica*.

## MATERIALS AND METHODS

One-year-old *F. carica* cv. Brown Turkey Modified-6 (BTM6) was a mother plant. A total of 540 stem cuttings were utilised as planting materials. The stem cuttings were grown in a glasshouse with three types of soil mixtures (sand and topsoil at 1:3 ratio [M1]; topsoil, peat and sawdust at 1:1:1 ratio [M2]; and peat and perlite at 1:1 ratio [M3]. The mixtures were placed in polybags of 6 cm × 9 cm in dimension. Meanwhile, two types of stem cuttings used were semi-hardwood (C1) and hardwood (C2). The treatment combination of planting medium and stem cutting was randomly assigned in a split-plot design of five replications. The planting medium and cutting type were treated as primary and subplot plants, respectively. The total of 18 experimental units per treatment per block were used in this experiment.

**Figure 1:**
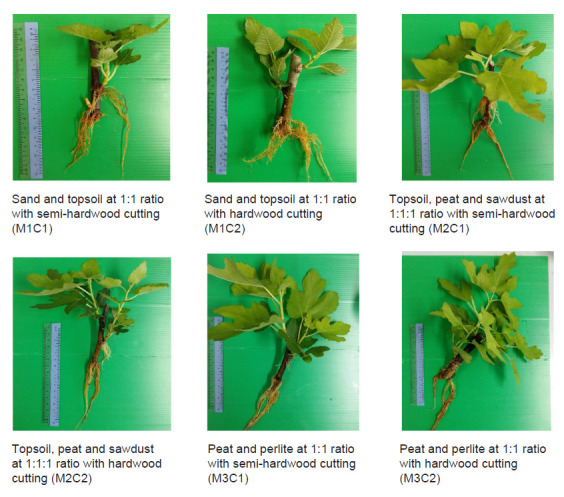
*F. carica* cv. BTM6 samples between treatments after 50 days.

Following 50 days of cultivation, the plant was harvested (Fig. 1). The parameters took into account were a total shoot fresh weight, a number of branches, leaves, newly emerged roots, primary roots, secondary roots, tertiary roots, in addition to a shoot length, shoot width, leaf length, leaf blade length, leaf blade width, petiole length, petiole width, root fresh and dry weight, the most extended primary root length, and the longest secondary root length. The total fresh weights were obtained right after the harvesting. The number of leaves were counted from every branch. The root dry weight was gained by oven dried the fresh sample at 45°C until it reaches constant weight. Number of newly emerge roots were taken from the number of small dots that appear on the cutting which later grow into the primary root.

Data were analysed by using analysis of variance (ANOVA) to test statistical significance among treatments. Subsequent multiple comparisons of means were performed employing the Duncan New Multiple Range Test (DNMRT). These statistical tests were conducted using the open-source Statistical Analysis Software (SAS) version 9.4 (SAS Institute, North Carolina, US).

## RESULTS AND DISCUSSION

There was a significant influence of the propagation medium on root dry weight, number of newly emerged roots, number of tertiary root and length of secondary root (Table 1). Based on the study, M3 showed a significantly higher root dry weight of 0.08 g compared to M2 (0.03 g) and M1 (0.02 g). It may be attributed to ample moisture and nutrient content of peat and excellent aeration properties of perlite. According to Waseem *et al.* (2013), a suitable propagation medium should be able to hold water and nutrient while at the same time has high porosity for root growth and development. Sufficient moisture needs to be available in the propagation medium to promote faster rooting of cutting (Hassanein 2013). In fact, cuttings might wilt and die if the rate of transpiration is higher than the rate of water absorption (Okunlola & Akinpetide 2016). Peat may supply sufficient amount of moisture and organic matter, thus, contributing to the early growth of cutting (Wahome *et al.* 2011). In addition, Samar and Saxena (2016) stated that perlite could be used to improve the aeration of the propagation medium. The previous study (Vâşcă *et al*. 2017) also recorded the highest percentage of rooting of *Abutilon hybridum* cutting propagated in perlite. Ali and Ahmad (2017) also reported that a higher rate of root length was recorded for the treatment with a combination of peat and perlite.

Nevertheless, the lowest performance of M1 might be attributed to the reduced capability to sustain water and nutrients. According to Razdan [2003, as cited in Okanlawon *et al.* (2016)], sands generally have weak water-holding and cation exchange capacities. Inadequate water supply will affect the nutrient and mineral transportation within the plant body. Therefore, a combination of peat and perlite (M3) in this study provided an optimum condition for cutting rooting due to high water holding capacity, organic matter and porosity that promotes higher root growth and development.

As for the type of cutting, hardwood cutting (C2) showed significantly higher root fresh weight (0.64 g), root dry weight (0.06 g), number of the tertiary root (209), length of primary root (10.37 cm), and length of secondary root (5.86 cm) as compared to semi-hardwood cutting (C1) of 0.35 g, 0.02 g, 65, 6.07 cm and 1.95 cm for each parameter, respectively (Table 2). However, other parameters, including a number of newly emerge root, a quantity of primary root and number of the secondary root, were not significant between cuttings. This result was concurrent with Souza *et al*. (2015), in which a higher increase in a number of roots and total root length was recorded in hardwood cutting. This could be attributed to higher nutrient and carbohydrate storage in hardwood cutting, resulting in a better root growth performance (Tchoundjeu & Leakey 1996; Wahab *et al*. 2001; Khapare *et al.* 2012). According to Hartmann *et al*. (2011), the reserve amount of carbohydrate in cuttings was correlated with plant survival. Ibironke (2019) reported that hardwood cuttings were ideally fit for use in *Bougainvillea* propagation as they showed a better growth performance compared to semi-hardwood cuttings.

In term of shoots, there was no significant difference among treatments except for a total shoot fresh weight (Tables 3 and 4). However, parallel result with root growth was recorded as the combination of peat and perlite (M3) showed significantly higher total shoot fresh weight of 6.15 g as compared with M2 (2.88 g) and M1 (1.52 g). This indicated that M3 significantly enhanced root and shoot growth of the fig cutting. The previous study by Okunlola and Akinpetide (2016) also observed that *Ficus benjamina*, grown in propagation medium containing a high amount of organic matter had a greater number of leaves owing to the abundance of essential nutrients for plant uptake. Peat provides proper aeration that helps increase the yield of plants (Olle *et al.* 2012). Canadian Sphagnum Peat Moss Association (CSPMA) (2014) also stated that functional cation exchange capacity (CEC) of peat helps to hold minerals and gradually release them in order to prevent fertilisers leaching. According to Robertson (1993) (as cited in Do & Scherer 2013), peat was considered as the best option to produce the best quality and yield for ornamental cultivation.

Nevertheless, hardwood cuttings showed a higher total shoot fresh weight (4.8 g) compared to semi-hardwood cuttings (2.2 g) (Table 4). Hartmann *et al.* (2011) mentioned that hardwood cutting was readily available as cutting material by removing leaves without damaging the bark. Furthermore, fig trees are often propagated via hardwood cuttings of one-year age plants (Pipattanawong *et al.* 2008). Okunlola (2013) mentioned that hardwood cuttings were firm and did not bend as easily as the wood of dormant mature stems. According to Darwesh [2000, as cited in Okunlola & Akinpetide (2016)], the success of woody stem cutting towards plant survival depends on the physiological state of the mother plant. Thus, hardwood cuttings promote an improved early growth performance relative to semi-hardwood due to a matured physiology structure.

## CONCLUSION

Based on this study, the survival rate of hardwood cuttings was the highest compared to semi-hardwood cuttings. The findings showed that 75 hardwood cutting survived when grown in a medium consisting of 50% perlite and 50% sand. Whereas, 51 semi-hardwood cuttings survived when grown in a medium consisting of sand and topsoil. It can be concluded that the early growth rate of *Ficus carica* increased by the usage of peat and perlite as propagation medium and hardwood cuttings as propagation materials. Besides, after 50 days, the plants developed a large number of leaves and roots and were stable for transplantation.

## Figures and Tables

**Table 1:** Mean of root growth of fig affected by propagation medium.

TRT/Parameters	RFW (g)	RDW (g)	NNER	NPR	NSR	NTR	LPRL (cm)	LSRL (cm)
M1	0.34^a^	0.02^b^	16.10^ab^	24.50^a^	214.70^a^	86.30^b^	9.13^a^	2.440^b^
M2	0.40^a^	0.03^ab^	31.60^a^	24.00^a^	151.30^a^	76.80^b^	6.44^a^	2.850^b^
M3	0.74^a^	0.08^a^	6.30^b^	34.70^a^	293.30^a^	249.20^a^	9.09^a^	6.420^a^

*Note*: Means followed by similar alphabetical letter in the same column are not significantly different at (*p* ≤ 0.05). RFW = root fresh weight; RDW = root dry weight; NNER = number of newly emerge root; NPR = number of primary root, NSR = number of secondary root; NTR = number of tertiary root; LPRL = length of longest primary root; LSRL = length of longest secondary root.

**Table 2:** Mean of root growth of fig affected by types of cutting.

TRT/Parameters	RFW (g)	RDW (g)	NNER	NPR	NSR	NTR	LPRL (cm)	LSRL (cm)
Semi-hardwood (C1)	0.35^b^	0.02^b^	12.07^a^	20.93^a^	157.13^a^	65.20^b^	6.07^b^	1.95^b^
Hardwood (C2)	0.64^a^	0.06^a^	23.93^a^	34.53^a^	282.40^a^	209.67^a^	10.37^a^	5.86^a^

*Note*: Refer Table 1 for the abbreviation.

**Table 3:** Mean of shoot growth of fig as affected by propagation medium.

TRT/Parameters	TSFW (g)	NOB	SL (cm)	SW (cm)	NL	LL (cm)	LBL (cm)	LBW (cm)	PL (cm)	PW (cm)
M1	1.52^b^	1.50^a^	2.49^a^	0.24^a^	6.80^a^	3.77^a^	2.68^a^	2.02^a^	1.09^a^	0.16^a^
M2	2.88^ab^	1.80^a^	4.19^a^	0.30^a^	9.30^a^	4.44^a^	3.09^a^	2.38^a^	1.35^a^	0.21^a^
M3	6.15^a^	1.60^a^	4.74^a^	0.32^a^	8.80^a^	4.82^a^	3.46^a^	2.78^a^	1.36^a^	0.16^a^

*Note*: Means followed by similar alphabetical letter in the same column are not significantly different at (*p* ≤ 0.05). TSFW = total shoot fresh weight; NOB = number of branches; SL = shoot length; SW = shoot width; NL = number of leaves; LL = leaf length; LBL = leaf blade length; LBW = leaf blade width; PL = petiole length; PW = petiole width.

**Table 4:** Mean of shoot growth of fig as affected by types of cutting.

TRT/Parameters	TSFW (g)	NOB	SL (cm)	SW (cm)	NL	LL (cm)	LBL (cm)	LBW (cm)	PL (cm)	PW (cm)
Semi-harwood (C1)	2.24^a^	1.47^a^	2.93^a^	0.27^a^	5.73^a^	4.17^a^	2.84^a^	2.25^a^	1.33^a^	0.17^a^
Hardwood (C2)	4.80^a^	1.80^a^	4.68^a^	0.31^a^	10.87^a^	4.52^a^	3.31^a^	2.53^a^	1.21^a^	0.19^a^

*Note*: Refer Table 3 for the abbreviation.
